# Unraveling the complexity of glycosphingolipidome: the key role of mass spectrometry in the structural analysis of glycosphingolipids

**DOI:** 10.1007/s00216-024-05475-7

**Published:** 2024-08-14

**Authors:** Karel Hořejší, Michal Holčapek

**Affiliations:** 1 Department of Analytical Chemistry, Faculty of Chemical Technology, University of Pardubice, Studentská 573, 53210 Pardubice, Czech Republic; 2 Department of Chemistry, Faculty of Science, University of South Bohemia in České Budějovice, Branišovská 1760, 370 05 České Budějovice, Czech Republic

**Keywords:** Glycosphingolipids, Mass spectrometry, Liquid chromatography, Structural elucidation, Fragmentation, Derivatization

## Abstract

Glycosphingolipids (GSL) are a highly heterogeneous class of lipids representing the majority of the sphingolipid category. GSL are fundamental constituents of cellular membranes that have key roles in various biological processes, such as cellular signaling, recognition, and adhesion. Understanding the structural complexity of GSL is pivotal for unraveling their functional significance in a biological context, specifically their crucial role in the pathophysiology of various diseases. Mass spectrometry (MS) has emerged as a versatile and indispensable tool for the structural elucidation of GSL enabling a deeper understanding of their complex molecular structures and their key roles in cellular dynamics and patholophysiology. Here, we provide a thorough overview of MS techniques tailored for the analysis of GSL, emphasizing their utility in probing GSL intricate structures to advance our understanding of the functional relevance of GSL in health and disease. The application of tandem MS using diverse fragmentation techniques, including novel ion activation methodologies, in studying glycan sequences, linkage positions, and fatty acid composition is extensively discussed. Finally, we address current challenges, such as the detection of low-abundance species and the interpretation of complex spectra, and offer insights into potential solutions and future directions by improving MS instrumentation for enhanced sensitivity and resolution, developing novel ionization techniques, or integrating MS with other analytical approaches for comprehensive GSL characterization.

## Introduction

**Fig. 1:**
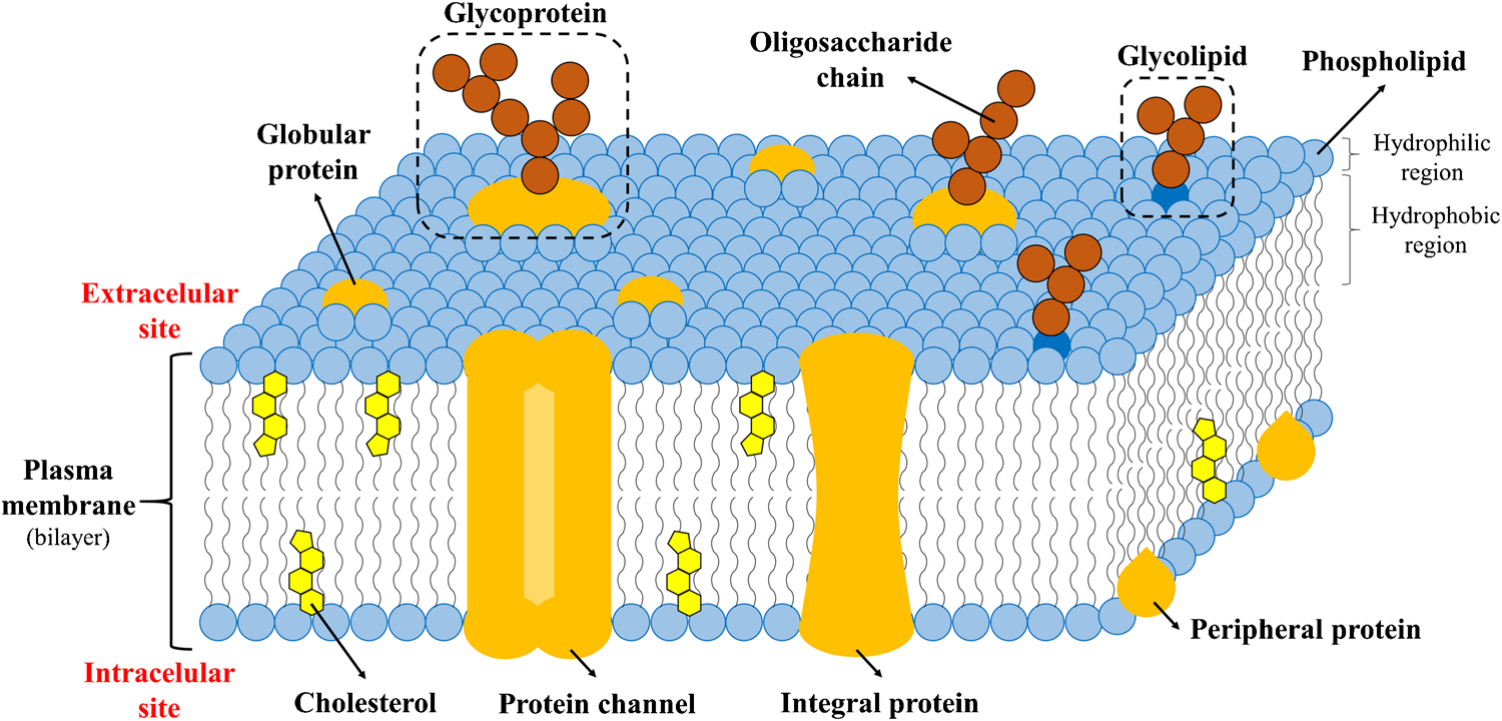
Cross-section and structure of plasma membrane

GSL comprise a vast group of remarkably heterogeneous biomolecules that are found in all eukaryotes, as well as some prokaryotes and viruses. GSL are ubiquitous membrane components, which are almost exclusively located on the outer leaflet of cell plasma membranes and in intracellular organelles (Fig. 1) [1, 2]. GSL are also found in body fluids, where they either circulate freely or are transported in lipoproteins [3]. GSL are amphiphilic molecules composed of two distinct parts. The hydrophobic region consists of a ceramide backbone anchored into the plasma membrane, while the hydrophilic region composed of a glycan moiety glycosidically linked to a ceramide backbone faces the extracellular environment [4].

GSL are classified based on their charges into neutral, acidic, and basic. Neutral GSL (nGSL) include cerebrosides (1 glycan unit) and globosides (≥ 2 glycan units), while acidic GSL (aGSL) are subdivided into sialic acid-containing GSL called gangliosides and sulfated GSL called sulfatides with sulfate group at C3 hydroxyl of Gal. In contrast, basic GSL are rare [2].

The rapid and direct structural elucidation of GSL and other lipids proves to be critical for studying their functional roles in many biological processes as well as the fundamental mechanism of their metabolism and pathogenesis in various diseases. MS-based methods using either atmospheric pressure ionization (API) or matrix-assisted laser desorption/ionization (MALDI) have emerged as powerful techniques for GSL profiling in the lipidomic analysis [5]. Although GSL can be relatively easily ionized and fragmented to product ions, providing information about the head groups (i.e., lipid class) and the type of the ceramide backbone, respectively fatty acyl composition (i.e., lipid species), the precise and in-depth characterization of GSL is quite challenging.

In this review, MS-based techniques tailored for the structural elucidation of GSL are discussed together with various fragmentation and ion activation techniques to study the glycan sequence, linkage positions, and fatty acid composition of GSL.

## Mass spectrometry characterization of GSL in biological samples

One of the current major challenges in lipidomics is the difficulty to separate and differentiate isomeric and isobaric species due to the immense structural variability in head groups, acyl chains, number and location of C = C bonds (cis/trans), and regioisomerism (*sn-*positions), which inhibit the delineation and assignment of their biological roles. The specific functions of the isomer have remained largely unknown due to these challenges [6]. In the case of GSL, the isomer problem is multiplied as many glycans have the same formula (e.g., Glc vs. Gal) as well as there can be distinct linkages in the oligosaccharide chain also with the option for either α- or β-glycosidic bonds, which further complicate GSL analysis [6, 7]. For instance, GlcCer and GalCer have different biological functions with GlcCer required for proper functioning of the epidermis, while GalCer maintains the structure and stability of myelin and the differentiation of oligodendrocytes. GlcCer and GalCer are also accumulated in various lysosomal storage disorders. Accumulation of GlcCer is a typical feature of Gaucher disease, while GalCer is accumulated in Krabbe disease. GlcCer has also been shown to be accumulate in neurodegenerative diseases such as Parkinson’s disease. However, little is still known about their role in tumor progression [8]. In general, a-series gangliosides are known to promote tumor growth while b-series gangliosides may have tumor-suppressive effects. Similarly, antibodies against GD1a and GD1b gangliosides are differentially expressed in various neurological and autoimmune diseases. Differentiation of GSL isomers is therefore crucial because of their distinct biological functions, disease associations, and therapeutic implications [9]. Moreover, the glycosphingolipidome is not only amazingly large, but also expanding with a number of new lipid species. Specifically, GlcCer with less prevalent α linkage rather than β linkage have been recently found [8, 10, 11] together with ceramides either lacking the 1-hydroxyl group [12] or having a fatty acyl attached to the 1-hydroxyl [13] alongside GSL with polyunsaturated very long-chain FA (C26–C36) [14]. Furthermore, humans only synthesize *cis* (Z) FAs, while *trans* (E) FAs are not endogenously produced but present in the human body due to dietary intake. They are well known to play an important role in various physiological processes and therefore, the separation of *cis/trans* isomers is of great interest. The coelution of different lipid subclasses can lead to ion suppression, obscuring the detection of low-abundant lipids [15]. To address these issues, improved separation of lipid subclasses and lipid species together with the ability to distinguish and identify GSL isomers is essential and highly advantageous for the investigation of their physiological role and functions in nature and disease [6, 15].

Several approaches are used to study GSL structures in connection with MS, such as direct infusion (DI), liquid chromatography (LC), supercritical fluid chromatography (SFC), mass spectrometry imaging (MSI), and ion mobility (IM).

### General workflows for the analysis of GSL

#### DI-MS

DI-MS (also termed shotgun lipidomics) is a technique when lipid extracts are directly introduced into the MS instrument without upfront separation [16]. The molecular characterization of lipid species relies either on the accurate *m/z* determination in the full scan mode or on the detection of specific fragmentation reactions in MS/MS experiments. In that case, the HRMS instruments are preferred due to their ability to differentiate isomeric and isobaric compounds [5, 17, 18]. Multi-dimensional MS-based shotgun lipidomics (MDMS-SL) allows the separation of many lipid (sub)classes through selective ionization of a certain category of lipids in the ion source (i.e., intra-source separation), even if the lipids are minor [16, 19, 20]. Although electrospray ionization (ESI) and MALDI are by far the most widely used ionization techniques in DI-MS, desorption electrospray ionization (DESI) [21], laser ablation electrospray ionization (LAESI) [22], and matrix-free laser desorption ionization (LDI) [23] have also been successfully applied. The major advantage of shotgun analysis is the reproducibility and relative high-throughput capability, allowing rapid acquisition of the full mass spectrum within seconds while providing similar sensitivity to LC/MS approaches, especially in coupling with nano-ESI [24]. On the contrary, the major drawbacks are the possible carry-over effect and susceptibility to ion suppression due to the presence of other major lipids or polar compounds (e.g., phospholipids, polar metabolites, and salts), which limits the ionization capacity and may even completely suppress the signals of minor or poorly ionizable GSL. Thorough sample preparation is required to ensure the removal of these interfering compounds. More detailed reviews on MS-based shotgun lipidomics can be read elsewhere [25, 26].

Sample preparation for shotgun lipidomics is very straightforward and generally involves simple extraction focusing on efficient total lipid extraction and minimal sample cleanup. The total lipid extract can be obtained commonly by chloroform/methanol-based liquid–liquid extraction such as of that Folch (CHCl_3_/CH_3_OH; 2:1, v/v) [27] and Bligh-Dyer (CHCl_3_/CH_3_OH; 1:2, v/v) [28]. In these protocols, lipids are partitioned into the lower chloroform phase. Polar solvent, such as methanol, ethanol, or ispropylalcohol, is used to increase the solubility of the lipids in the organic phase [29, 30]. These biphasic systems are able to recover wide range of lipids; however, sialylated and sulfated GSL or neutral GSL with at least four glycan residues mostly partition to the methanol-rich layer. On the contrary, neutral GSL with less than four glycan residues and other less polar lipids remain rather in the chloroform-rich layer. Thus, these methods do not provide effective recovery of the amphiphilic and highly polar GSL, as they generally require more aqueous portion [29, 31]. Over the years, several modifications and alternative strategies to these original protocols have been developed. One of them is the method described by Matysh et al*.* [17], which utilizes the mixture of methyl tert-butyl ether (MTBE) and methanol in ratio 10:3. This method was specifically developed for shotgun lipidomics of samples with excessive amounts of biological matrices. Furthermore, single-phase butanol-methanol (BUME) extraction system firstly described by Löfgren et al*.* (n-butanol/CH_3_OH; 3:1, v/v) [32, 33] and further modified by Alshehry et al*.* (n-butanol/CH_3_OH; 1:1, v/v) [34] has been reported to provide a similar yield of lipids compared to traditional Folch and Bligh-Dyer methods. Over the past years, monophasic extractions, also termed as protein precipitation methods, have gained popularity and have been applied to the simultaneous analysis of polar and non-polar lipids and other metabolites. The one-phase extraction methods are generally faster, cheaper, and less complex compared to conventional two-phase partition systems, and eliminate the risk of losses during transfer between phases; however, they do not allow the removal of polar and ionic impurities, leading to an increased risk of matrix effects and ion suppression. Thus, the application of one-phase extractions should be limited to polar lipid classes or should be followed by a sample cleanup using liquid–liquid extraction (LLE) and/or solid-phase extraction (SPE) [5, 35, 36]. The one-phase extraction is usually achieved through simultaneous protein precipitation with a variety of organic solvents including methanol, ethanol, acetonitrile, acetone, isopropanol, n-butanol, as well as their mixtures [35–38].

#### LC/MS

LC/MS is a key, well-established, and powerful analytical method used in lipidomics, which allows lipid subclass and/or lipid species separation when coupled to MS. The most frequently used ionization technique in LC/MS-based lipidomics is ESI, which is best suited for a wide range of lipids, including GSL due to several significant advantages including high sensitivity, easy coupling with chromatographic techniques, and structural details based on the use of tandem mass spectrometry (MS/MS) with high mass accuracy. In contrast, APCI and APPI are valuable alternatives for less polar lipids [5, 39]. The initial lipid extraction for the lipidomic analysis using LC–MS methods is similar to those described in the “DI-MS” section, i.e., using organic solvents. In contrast to sample preparation used for DI-MS, the sample preparation for LC–MS often requires more rigorous sample cleanup, including SPE to further purify the lipid extract by removing impurities and concentrating the lipids [30]. If needed, depletion of highly abundant lipids such as glycerolipids and phospholipids can be performed using alkaline hydrolysis [40] or special ZrO_2_/TiO_2_-based SPE method can be employed for removal of phospholipids to allow the analysis of low-abundant lipid species [41]. In addition, SPE or open column chromatography can be used to fractionate the lipid extract into subfractions [40].

#### MSI

MSI has become a popular and powerful method perfectly designed for the analysis of solid samples (e.g., tissues) with the ability to simultaneously display both spatial distribution and molecular level information. The most frequently used ionization technique employed in MSI is MALDI, but other ionization techniques could be employed as well, e.g., DESI, LAESI, or secondary ion mass spectrometry (SIMS) [42, 43]. The major advantages of MALDI are minimal sample preparation, high tolerance to salts, and the ability to relatively easily ionize heavily glycosylated GSL, but with lower ionization efficiency compared to ESI [29]. MALDI also has a few limitations, such as the inability to resolve isomers without prior separation and generally high background noise and ion suppression effects due to the formation of matrix clusters [29, 44].

The most widely used MSI technology applied for rapid in situ screening and mapping the spatial distribution of individual lipid species in biological samples is MALDI coupled to time-of-flight analyzers (MALDI-TOF). However, the comprehensive analysis is limited since the MSI is largely based on the qualitative comparison of healthy and diseased samples [45–47]. The coupling of MSI with Orbitrap or ion cyclotron resonance has provided deeper insight into the lipidomic complexity of biological samples [48], such as the application of MALDI-Orbitrap using MS/MS spectra to facilitate structural elucidation of even highly complex sulfo-GSL with up to five hexose moieties [49]. MSI techniques generally require minimal sample preparation. In MALDI, the tissue samples are first cryodissected into slices (~ µm), placed on a target surface, co-crystallized and immobilized with a suitable matrix, and then irradiated by laser to produce ions [29]. Common matrices used for GSL analysis include, for example, 2,5-dihydroxybenzoic acid, 1,5-diaminonaphtalene, 4-hydrazinobenzoic acid, 6-aza-2-thiothymine, 6,7-hydroxycoumarin (esculetin), and α-cyano-4-hydroxycinnamic acid [29, 50]. MALDI matrices used in lipidomics were also well-reviewed by Leopold et al. [51].

##### Ion mobility

In recent years, IM has appealed as a suitable technique for the separation of lipid isomers. However, due to the current resolving power limitations, lipid isomers cannot be fully resolved by IM alone in complex mixtures [6], which led to the interfacing of IM with LC/MS with great potential for the separation of lipid isomers together with increased selectivity and sensitivity [52].

Wojcik et al. [6] utilized ultrahigh-resolution IM separation with traveling waves in a serpentine and extended multipass SLIM platform for selected lipid and glycolipid isomers. They achieved partial separation of GlcSph 18:1;O2 *vs.* GalSph 18:1;O2 and GlcCer 18:1;O2/18:0 *vs.* GalCer 18:1;O2/18:0, differing only in the identity of glycan, which was achieved after four passes (~ 60 m path). Moreover, the baseline separation of GD1a and GD1b gangliosides, which only differ in the location of sialic acid residues, has been accomplished even with a minimal possible path of 1.25 m (i.e., without using multipass separation). However, the major issue is the limited number of passes due to increasing peak widths with the increasing number of passes, which reduces the detection window and range of mobilities. May et al*.* [53] also resolved GD1a and GD1b gangliosides in a standard mixture as doubly sodiated species [M + 2Na]^2+^ along with two pentasaccharide GSL differing in the location and linkage of fucose. Djambazova et al*.* [54] have reported a partial separation of GD1a and GD1b isomers with 36:1;O2 and 38:1;O2 ceramide in tissue samples using MALDI-TIMS. Xu et al*.* [55] have shown an effective resolution of GlcCer and GalCer species from human plasma and cerebrospinal fluids using differential mobility spectrometry coupled to LC/ESI–MS. Sample preparation for IM is similar to DI-MS or LC–MS.

In summary, although the separation and characterization of lipid isomers still remain very challenging and only a limited number of lipidomic studies have been carried out to differentiate GSL isomers using IM technologies, recent advances in chromatography and IM through novel instrumental developments have pushed the popularity of IM forward by enhancing its resolution and sensitivity. The coupling of IM with LC/MS is thus likely to become a very valuable tool capable of efficiently separating and reliably distinguishing various GSL isomers. However, further progress is still needed as potential applications of IS are still being discovered [56].

### Structural elucidation of neutral GSL

The MS/MS analysis of GSL relies on various dissociation techniques. Each dissociation technique provides a distinct level of structural information since it cleaves bonds at different locations of the molecule. Their combination can provide complementary structural details of GSL [29].

**Fig. 2:**
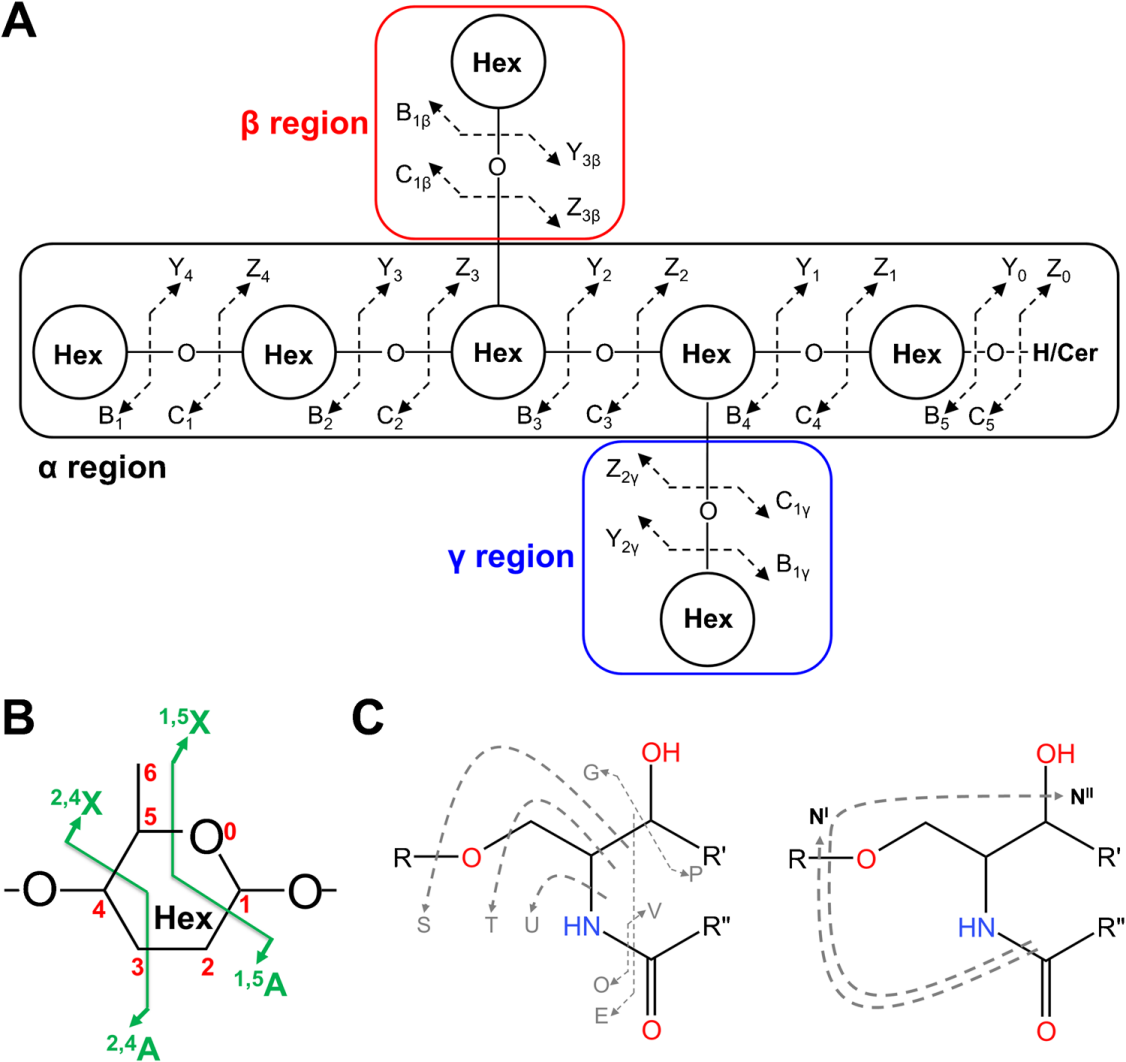
Fragmentation patterns of GSL (adopted from [57, 58, 123]); Hex refers to hexose

Biological functions of GSL, as well as other lipids, highly depend on their varying expression levels and structural diversity, including carbon–carbon DB locations, *cis/trans* isomerism, and the *sn*-position of the fatty acyl chain(s), which complicate the structural elucidation. The MS/MS analysis is essential in the structural elucidation of GSL. The systematic nomenclature of MS/MS fragments for the carbohydrate part of GSL was proposed by Domon and Costello [57] and includes fragments containing the non-reducing end (i.e., A, B, and C) and the reducing end (i.e., X, Y, and Z). Fragments B, C, Y, and Z correspond to glycosidic cleavages that determine the glycan sequence (Fig. 2A), while A and X fragments are cross-ring cleavages allowing the differentiation of linkage positions (Fig. 2B). Since A, B, and C ions do not include ceramide structure, unlike their X, Y, and Z counterparts, their masses are not affected by the lipid moiety. The nomenclature was later updated by Ann and Adams [58] in order to include more detailed ceramide fragments. The major fragments are shown in Fig. 2C, where the N^I^ and N^II^ fragments are diagnostic ions of the long-chain base of the ceramide moiety. It implies that the fragmentation pathways of GSL can be predicted and structural databases can be constructed in silico to identify GSL by matching MS/MS spectra with the database, which is an indisputable advantage in the structural analysis of GSL.

Neutral GSL are relatively poorly ionized in the negative ion mode due to their basic (i.e., amino glycan-containing) and acidic counterparts [59]; thus, neutral GSL are commonly analyzed in the positive ion mode, where GSL are better ionized and provide abundant Y/Z-pair ion series indicative of sequence information accompanied by less common B/C-type fragments. A/X-type ions involving C–C bond usually require higher energies [60].

#### Simple neutral GSL (up to 4 monosaccharide units)

Collision-mediated dissociation is a conventional dissociation technique employed for MS/MS experiments. Both low-energy collision-induced dissociation (CID) and higher-energy collisional dissociation (HCD) provide structural information for elucidating the glycan sequence in the positive ion mode (i.e., sequential cleavage of a monosaccharide unit represented by a series of Z/Y-type ions) or in the negative ion mode (i.e., B- and C-type ions) and ceramide moiety of GSL. An alternative implemented exclusively for ion trap MS is pulsed Q dissociation (PQD), which deposits higher energies on the ions compared to CID and allows the observation of low *m/z* fragments that are usually excluded from CID, however at the cost of reduced fragmentation efficiency [29, 61, 62].

Incremental Δ*m/z* indicates the loss of a hexose (Δ*m/z* 162) and N-acetlyhexosamine (Δ*m/z* 204) [29]. In addition, the ceramide composition, respectively, sphingoid bases, can be identified from the specific fragment ions in the positive mode (Table 1) [62].

**Table 1:** Typical N^II^ fragments corresponding to the respective sphingoid base [62]

*m/z*	238	236	266	264	262	294	292	282
Base	16:0;O2	16:1;O2	18:0;O2	18:1;O2	18:2;O2	20:0;O2	20:1;O2	18:0;O3

**Fig. 3:**
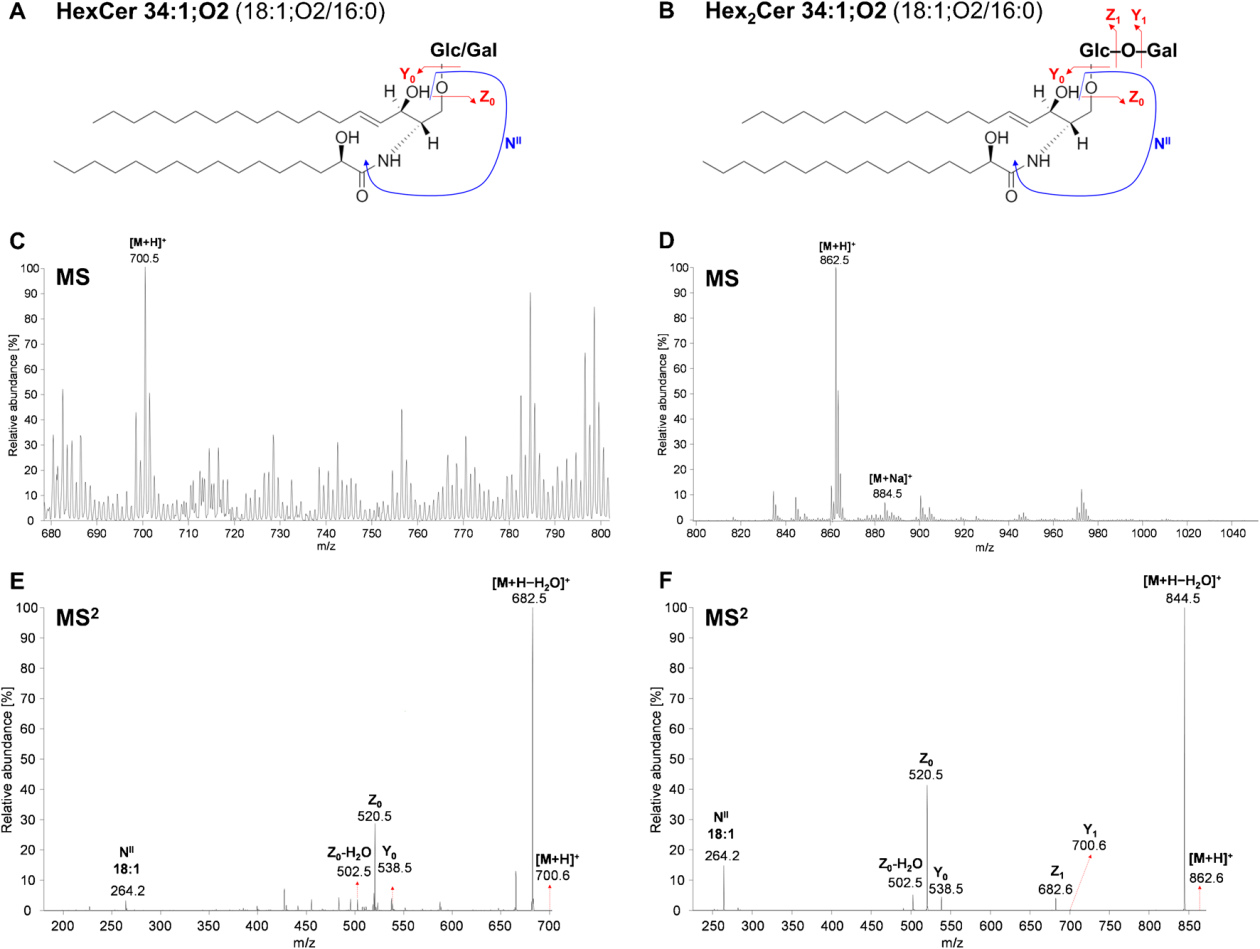
Examples of fragmentation behavior of (**A**) HexCer and (**B**) Hex2Cer. MS spectra of (**C**) HexCer and (**D**) Hex2Cer, and MS^2^ spectra of (**E**) HexCer 34:1;O2 and (**F**) Hex2Cer 34:1;O2 with characteristic fragment ions obtained in human plasma analyzed by HILIC/ESI–MS/MS

**Fig. 4:**
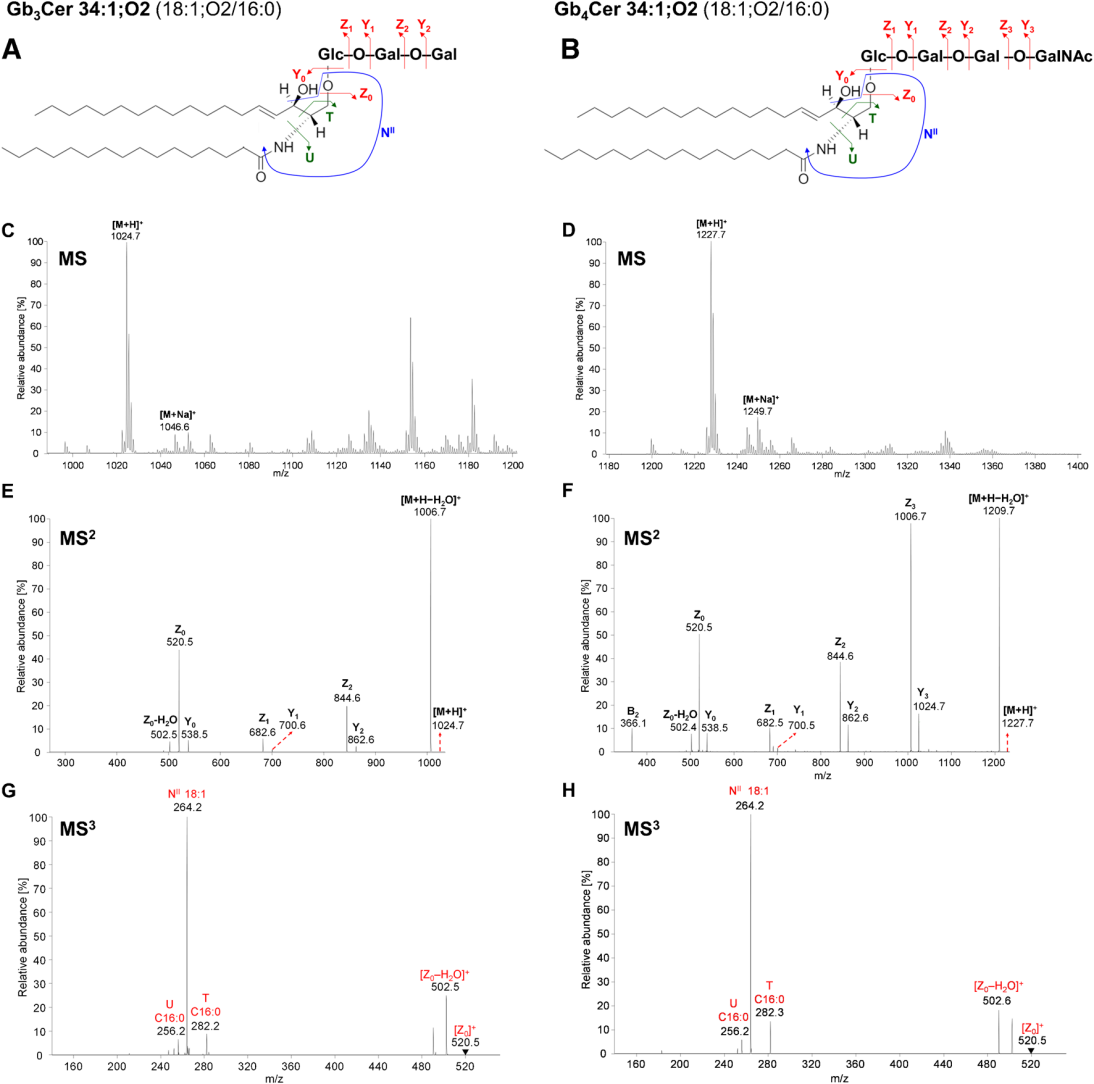
Examples of fragmentation behavior of (**A**) Gb3Cer and (**B**) Gb4Cer, MS spectra of (**C**) Gb3Cer and (**D**) Gb4Cer, MS^2^ spectra of (**E**) Gb3Cer 34:1;O2 and (**F**) Gb4Cer 34:1;O2. MS^3^ spectra of the respective [Z_0_]^+^ ion of (**E**) Gb3Cer and (**F**) Gb4Cer together with characteristic fragment ions obtained in human plasma analyzed by HILIC/ESI–MS/MS

General fragmentation patterns of simple neutral GSL using CID have previously been extensively studied [62–64], as illustrated by examples in Figs. 3 and 4.

To distinguish isomers with subtle structural differences, multistage collisional dissociation has to be usually used, even when coupled with the chromatographic separation. Li et al*.* [65] showed that MS^n^ of permethylated GSL allowed the differentiation of Gb_4_Cer (specific ion at *m/z* 315) and iGb_4_Cer (specific ion at *m/z* 357).

Whereas collision-mediated dissociation methods mainly induce glycosidic bond cleavage, ultraviolet photodissociation (UVPD) yields extensive fragmentation patterns of GSL to study neutral and acidic GSL [66], which provides more informative cross-ring fragments of the glycan moieties (A/X-type) and several unique UVPD-specific cleavages at ceramide C–C and C–N bonds that allow differentiation between isomeric glycans and ceramides. In particular, UVPD can reliably locate C = C bonds in the ceramide moieties of GSL. Despite the advantages, UVPD is not widely used due to the lack of automated software tools for spectra annotation; however, there are a few lipidomics applications [61, 66, 67]. For instance, Ryan et al*.* [68] have reported that 193-nm UV irradiation leads to the extensive fragmentation of both glycan and ceramide parts in neutral GSL and gangliosides along with a certain level of fragmentation close to carbon–carbon double bonds (DB). The analysis of C = C bond location by UVPD is limited due to the low fragmentation efficiency caused by low photon absorption by C = C bond; therefore, a high-power laser or incorporation of more efficient chromophores is needed to improve the sensitivity to locate C = C bonds [69].

In addition, Pham et al*.* [70] demonstrated the discrimination of GSL epimers, namely, GlcCer/GalCer and GlcSph/GalSph, using a unique fragmentation technique called radical-directed dissociation. The discrimination was based on the inverted abundance of the major fragments caused by the differential ability of GlcCer and GalCer to lose water (i.e., neutral loss of water is easier in Gal than in Glc). However, the application of radical-directed dissociation in the GSL analysis is very limited, mainly due to the complicated sample preparation.

#### Complex neutral GSL (more than 4 monosaccharide units)

The analysis of complex and heavily glycosylated GSL poses an additional challenge as they suffer from poor ionization efficiency and multistage fragmentation mass spectrometry (MS^n^) is usually required to achieve step-by-step cleavage of the glycosidic bonds and ceramide backbones. It is known that ESI sensitivity decreases with the increasing length of the glycan chain of GSL due to increased hydrophilicity [59, 71].

Furthermore, a large majority of GSL share Gal–Glc disaccharide core linked to the ceramide moiety, which may complicate the product spectral analysis because the Y_0_/Z_0_, Y_1_/Z_1_, and Y_2_/Z_2_ fragments of different GSL species may have the same mass, despite their intensities vary among GSL species [60]. GSL containing 2-hydroxy fatty acyl groups are also typically characterized by the abundant fragment ion derived from the loss of the hydroxy-acyl group [72].

In some cases, the analysis of underivatized GSL-derived oligosaccharides in the negative ion mode may be advantageous for isomer recognition due to low chemical background noise and low level of cation adduct formation [73, 74]. Negative ion MS/MS spectra of oligosaccharides are generally dominated by a series of B/C-type ions providing information about the glycan sequence. More interestingly, C_1_ ion can provide additional information about the terminal Gal linked to GlcNAc. The fragmentation of Gal1–4GlcNAc linkage is more facile and thus can be readily cleaved, while Gal1–3GlcNAc linkage is more resistant. However, when Gal is substituted with Fuc, the 1–3 linkage can be cleaved (Table 2) [73].

**Table 2:**
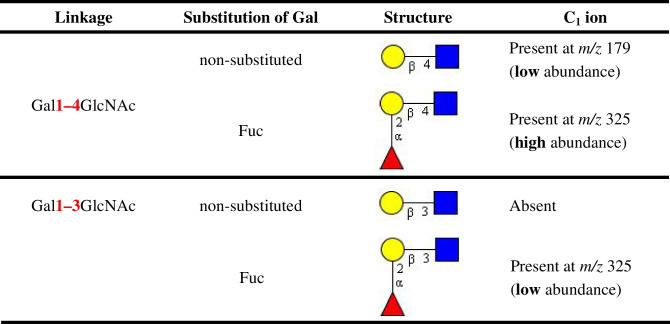
Distinction of 3-/4-linked Gal to GlcNAc with and without Fuc substitution [73]

Legend: Yellow circle refers to galactose (Gal), blue square refers to N-acetylglucosamine (GlcNAc), red triangle refers to fucose (Fuc)

Generally, cross-ring fragmentation (i.e., A-type ions) is useful for defining the linkage positions between individual monosaccharides [74]. The cross-ring ^0,2^A-type cleavage is typical for 4-linked GlcNAc or Glc (i.e., type 2 chain), whereas it is not produced for 3-linked GlcNAc (i.e., type 1 chain). It can be used to discriminate between Gb and iGb. In addition, the double glycosidic D-type cleavage (i.e., C–Z double cleavage) is unique to non-substituted 3-linked GlcNAc/Glc or 4-linked GlcNAc/Glc substituted with Fuc (Table 3). Similarly, D-type ions indicate 3-linked GlcNAc substituted with Gal at the 4-position. Taken together, A- and D-type ions are important for the differentiation of type 1/2 chain, and blood group H, Le^a^, Le^b^, Le^x^, and Le^y^ determinants [73].

**Table 3:**
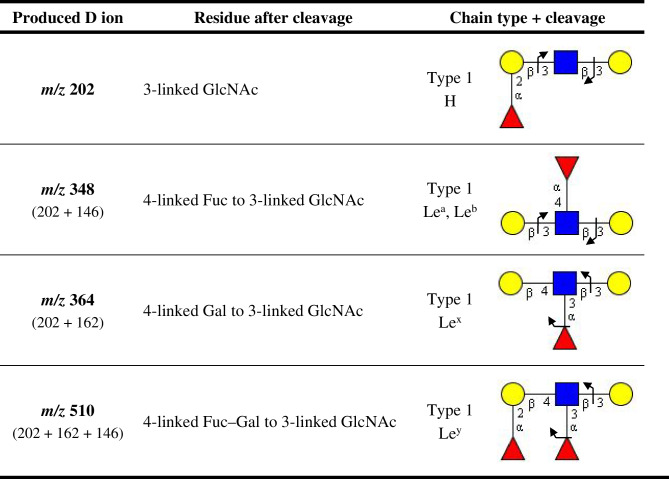
Typical D-type fragments of type 1/2 chain and blood group determinants [73]

Legend: Yellow circle refers to galactose (Gal), blue square refers to N-acetylglucosamine (GlcNAc), red triangle refers to fucose (Fuc)

**Fig. 5:**
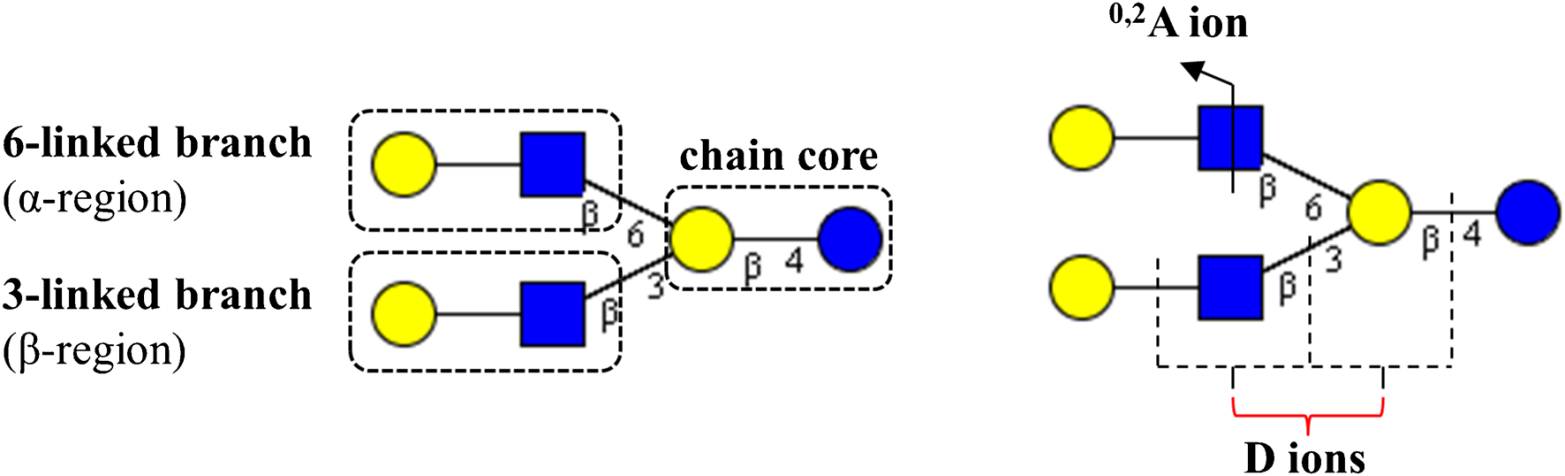
Scheme of 3- and 6-branched oligosaccharides with typical fragment ions [74]

In case of complex branched GSL-derived oligosaccharides, MS/MS spectra can provide complementary structural information. In MS/MS spectra of [M–H]^–^, fragment ions derived from 6-linked branches (α) are dominant, while those from 3-linked branches (β) are absent. In contrast, fragment ions from both branches are dominant in MS/MS spectra of [M–2H]^2–^. Similarly, double cleavage (D-type ion) occurs only at 3-linked branches (Fig. 5) [74].

In-depth description of the MS/MS of oligosaccharides can be found in this review [59]. The differentiation of blood groups A, B, and H, and Lewis blood group Le^a^, Le^b^, Le^x^, and Le^y^ determinants on GSL-derived oligosaccharides has been described in detail [75, 76].

### Structural elucidation of acidic GSL

In contrast, acidic GSL (i.e., sulfatides and gangliosides) are broadly analyzed in the negative ion mode since the molecule is readily ionizable due to anionic sulfate groups or sialic acid residues [77]. However, the application of positive ion mode is also possible [60]. GSL with acidic residues usually face in-source or post-source fragmentation, or produce metastable ions, especially when MALDI-MS is used [71, 72].

#### Sulfatides

**Fig. 6:**
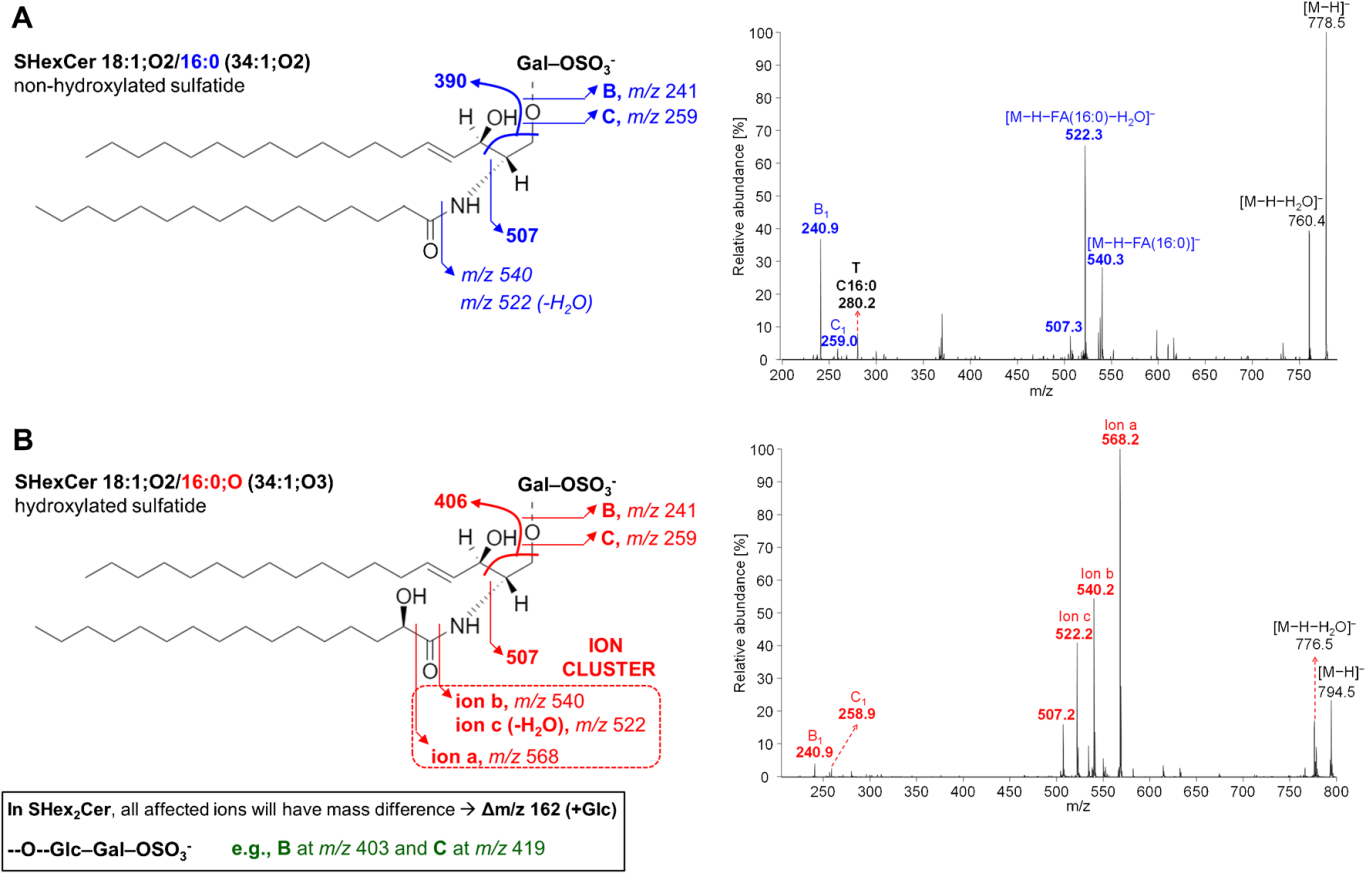
Characteristic fragmentation of non-hydroxylated and hydroxylated sulfatides in negative ion MS/MS [78]

Sulfatides provide a prominent diagnostic ion at *m/z* 97 (i.e., HSO_4_^−^ ion) [29], which is, however, not observed in ion trap MS because of the low mass cutoff, together with B/C-type fragment ions reflecting the 3-sulfoGal (*m/z* 259 and 241, for SHexCer) and 3-sulfoGal–Glc (*m/z* 419 and 403, for SHex2Cer) residues accompanied by the dehydration. Furthermore, the differentiation of non-hydroxylated and hydroxylated sulfatides has been well documented. The α-hydroxylated fatty acid-containing sulfatides are recognized by the unique and prominent ion cluster originated from the primary cleavage of the fatty acyl CO–CH(OH) bond (ion a) accompanied by the direct loss of fatty acyl as a ketene from the precursor ion via the NH–CO bond cleavage (ion b), which further undergoes the water loss (ion c) [77–79]. The difference between MS/MS spectra of non-hydroxylated and hydroxylated sulfatides is illustrated in Fig. 6.

#### Gangliosides

In general, gangliosides may be multiply charged, e.g., [M-H]^−^, [M-2H]^2−^, and [M-3H]^3−^, depending primarily on the number of sialic acids (NeuAc) and on the pH value, which influences the dissociation states [80]. Gangliosides have been considered difficult to analyze as sialic acid is relatively labile and preferentially lost during the ionization process. The neutral loss of sialic acid (*m/z* 290) [29] or additionally acetylated sialic acid (*m/z 332*) is favored when two or more sialic acids are present, but the stability can be improved by the formation of metal ions (i.e., [M–2H + Na]^−^). This is primarily a typical feature of MALDI-MS [72, 81]. The presence of two or more adjacent sialic acids is identified by fragment ions at *m/z* 581 (i.e., NeuAc–NeuAc), *m/z* 623 (i.e., NeuAc–NeuAc_2_), or *m/z* 914 (i.e., NeuAc–NeuAc–NeuAc_2_). This predictable fragmentation behavior allows sequencing of the oligosaccharide part of gangliosides and therefore, allows the distinction of the type of branches, i.e., a-, b-, and c-series gangliosides [80]. Generally, sialic acid glycoconjugates can be linked to Gal via 2–3/6 linkages and to GalNAc via 2–6 linkage. There are also α-/β-anomers of sialic acid with α-anomeric form being the most common, while β-anomeric form is typical for free sialic acid. It is also unclear if the β-anomer is present in oligosaccharide chains or ignored/escaped from detection due to low concentration. The linkage positions and anomeric configurations of sialic acid are reflected in the stability of the molecule during the ionization: β2–3 > α2–6 > α2–3 > β2–6 as well as in the characteristic fragmentation patterns in MS/MS spectra (Table 4) [81].

**Table 4:** Differentiation of 2–3/6 and α/β linkages of sialylated oligosaccharides [81]

Linkage and anomeric configuration		Characteristic fragments	
2–3-linked NeuAc (SA)	α linkage	^2,4^A-CO_2_; B_2_-CO_2_	
	β linkage		^2,4^A; B_1_-CO_2_
2–6-linked NeuAc (SA)	α linkage	^0,4^A-CO_2_; ^0,2^A, ^2,4^A	
	β linkage		^0,4^A
D-type ion at *m/z* 493	Internal location of SA on the 3-linked GlcNAc and Glc		

Legend: SA refers to sialic acid or also N-acetylneuraminic acid (NeuAc)

### Novel dissociation techniques for structural analysis of GSL

Nowadays, collision-induced dissociation (CID) MS/MS is a well-established dissociation technique providing the head group and ceramide composition. However, the ceramide backbones of GSL as well as other lipids may contain unsaturated FA with one or more carbon–carbon DB (C = C), which cannot be located using collision-mediated techniques alone. Since the structural elucidation of C = C bond location, *cis/trans* isomers, and *sn*-position isomers has recently become a hot topic, several novel and selective ion activation technologies have been developed to address this issue [82, 83].

#### Paternò-Büchi reaction

In 2014, Ma and Xia [84] introduced a novel method of pinpointing C = C bond locations by online coupling of the Paternò-Büchi (PB) reaction with MS/MS. The reaction is fast and highly specific, providing highly abundant diagnostic ions enabling confident localization of C = C bonds [85]. This strategy has also been applied for the large-scale analysis of C = C location isomers of different lipid subclasses in a variety of biological samples; however, it has a potential for the location of C = C bonds in ceramide moieties of GSL as well [86, 87]. Additionally, Bednařík et al*.* [88] developed an on-tissue PB reaction for the localization of C = C bonds in phospholipids and glycolipids by MALDI-MS using benzaldehyde.

#### Ozone-induced dissociation

Ozone-induced dissociation (OzID) was firstly implemented in 2008 by Blanksby’s group, who replaced inert collision gas in the mass spectrometer with O_3_/O_2_ mixture so that the ozonolysis can occur inside the collision cell [89]. OzID is a gas-phase reaction based on cycloaddition of O_3_ to unsaturated lipids generating metastable ozonide that spontaneously decays to more stable Criegee and aldehyde diagnostic ions with constant mass differences of 16 m*/z*. When coupled to soft ionization techniques, the method is highly specific and efficient for assignment not only the position of C = C bonds, but also their stereochemistry in unsaturated lipids [69, 90]. The major drawbacks are the need for modification of commercial instruments and specialized equipment generating ozone together with the longer reaction time (0.2–10 s) needed to accumulate detectable diagnostic ions due to the low ozone density allowed in the ion trap analyzer [85]. This reduces the analysis speed and makes the coupling with HPLC inefficient, which greatly restricts the application of OzID. To circumvent this limitation, OzID has been implemented in a high-pressure IM cell with high O_3_ density, significantly accelerating ozonolysis and producing abundant C = C fragment ions [91], which also facilitated a coupling with LC, a novel platform for the analysis of isomers [92].

For instance, Barrientos and coworkers have demonstrated the structural analysis of sodiated adduct ions of unsaturated GSL using OzID-MS that yielded more informative cross-ring cleavages compared to protonated ions [93]. Later, they reported that adducts can remarkably influence both the bond cleavage and the fragmentation behavior and illustrated distinct fragmentation patterns of [M + H]^+^, [M + Na]^+^, and [M + Li]^+^ precursor ions of GSL in OzID-MS. Specifically, they reported that [M + H]^+^ ion primarily undergoes dehydration yielding [M + H-H_2_O]^+^ ions followed by the sequential loss of monosaccharide units, while [M + Na]^+^ and [M + Li]^+^ adducts dissociate preferably at the DB yielding similar fragmentation patterns, albeit the relative intensities of diagnostic ions were remarkably different [94].

#### Epoxidation

**Fig. 7:**
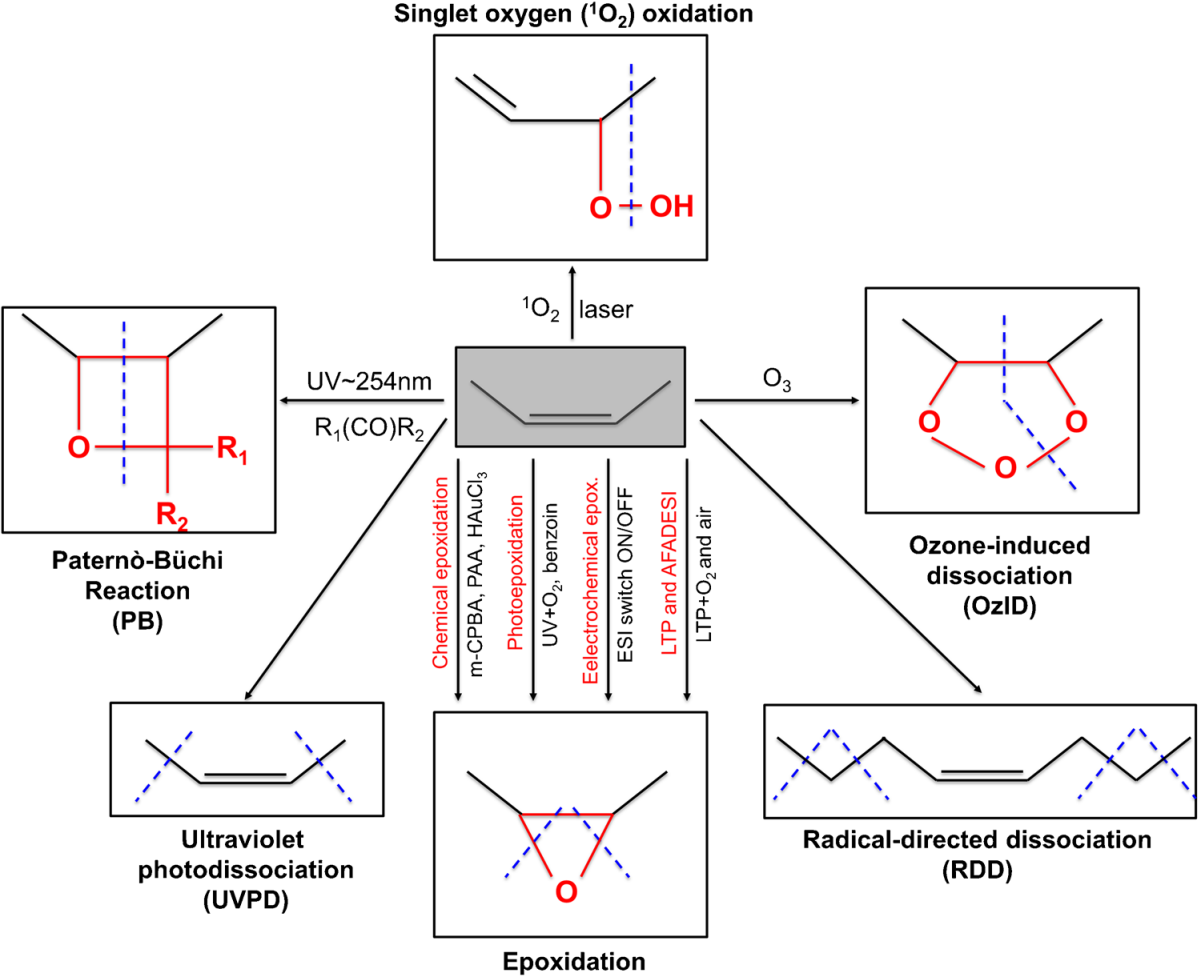
Schematic representation of various approaches utilized for the location of C = C bonds (modified from [69, 85, 100])

Recently, several innovative epoxidation reactions have been developed. Although the majority of them have been used for the identification of C = C bonds in other lipids than GSL. These methods can readily be applied for the differentiation of GSL isomers containing unsaturated fatty acyls as well. For example, the epoxidation using metachloroperoxybenzoic acid (m-CPBA) [95] and peracetic acid (PAA) [96] has been used for large-scale identification and spatial mapping of biological C = C isomers. Epoxidation is initiated by the oxidation of unsaturated lipids via m-CPBA or PAA either in solution or on-tissue reaction to generate an epoxide product, which is further subjected to CID-MS/MS analysis generating a pair of diagnostic ions pinpointing the location of C = C bond [96]. Specifically, the m-CPBA epoxidation is completed within minutes and without overoxidized by-products, showing the potential for high-throughput analysis [97] and making the epoxidation a versatile and user-friendly platform with minimal requirements for instrumentation [95]. Recently, Zhang et al*.* [98] proposed a rapid light-controlled photoepoxidation using benzoin to locate the positions of C = C in various isomers of unsaturated lipids in mouse tissue extracts, in both positive and negative ion modes. Moreover, the rapid switch-on/off electrochemical epoxidation controlled simply by tuning the electrospray voltage was recently developed to locate the C = C bond positions in lipids [99]. Furthermore, chloroauric acid (HAuCl_4_)-doped solvent introduced into an electrospray has been reported to induce epoxidation [100]. Next, low-temperature plasma (LTP)-induced epoxidation using atmospheric oxygen can also be used for the assignment of C = C bonds. The reaction is performed by blowing the LTP into the mixture of lipids in acetone/water (50/50, v/v) followed by rapid and nearly complete conversion of C = C bonds to the corresponding epoxides [101]. A novel method based on ambient oxidation coupled with air flow-assisted desorption electrospray ionization (AFADESI) was proposed to conveniently and rapidly characterize the spatial distribution of unsaturated lipid isomers using MSI technology [102]. A novel, online, and selective photosensitized oxidation of C = C bonds induced by singlet oxygen (^1^O_2_) coupled to CID-MS/MS has been proposed to distinguish positional isomers based on the formation of unique lipid hydroperoxide products (neutral losses) generated promptly after laser irradiation. Characteristic neutral losses arise from cleavage at the location of the hydroperoxide group of the respective lipid hydroperoxide, which was produced by the interaction of unsaturated lipids with ^1^O_2_ [103]. Various approaches used for the location of C = C bonds are summarized in Fig. 7.

**Fig. 8:**
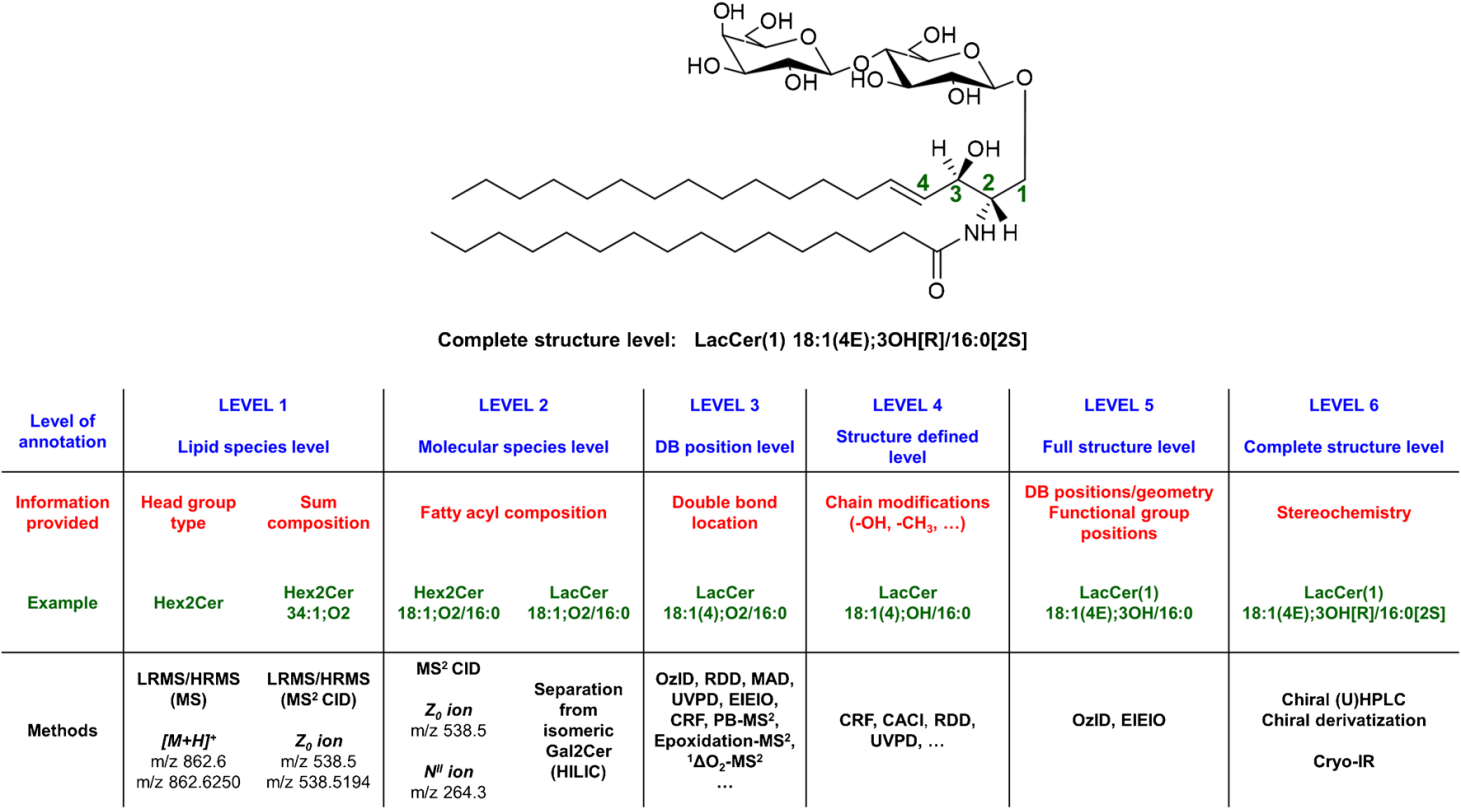
Diagram illustrating the different levels of structural characterization of GSL (modified from [69]). The sn-position level is not used for GSL structures as it is more pronounced in glycolipids and glycerophospholipids. The identified structural levels are named according to Liebisch et al. [124]. Legend: OzID, ozone-induced dissociation; RDD, radical-directed dissociation; MAD, metastable atom-activated dissociation; UVPD, ultraviolet photodissociation; EIEIO, electron impact excitation of ions from organics; CRF, charge-remote fragmentation; CACI, covalent-adduct chemical ionization; IR, infrared spectroscopy

In summary, CID commonly generates diagnostic ions of the glycan chain, sphingoid base, or N-fatty acyls composition, while more detailed structural information including branching and linkages in the glycan sequence and DB positions in the ceramide backbone can rather be determined using more specialized dissociation techniques (i.e., ECD, EDD, ETD, UVPD, RDD, OzID, ^1^O_2_, PB reaction, or epoxidation) [29]. Among the above-mentioned strategies, the PB reaction is the most commonly used method to locate C = C positions in both DI-MS/MS and HPLC/MS/MS workflows, although OzID and epoxidation provide much higher specificity. A more detailed description of the applications of AIMS techniques utilizing novel ion activation methods in the elucidation of unsaturated lipids can be found in the following reviews [69, 82]. Different levels of structural characterization of GSL obtained by different methods are summarized in Fig. 8.

### Deciphering GSL structures using chemical derivatization

In addition to MS-based techniques, the chemical derivatization of specific functional groups has a great potential to overcome the analytical barriers that still exist in the differentiation of isomeric and/or isobaric GSL [104]. The derivatization can increase the ionization efficiency of neutral and heavily glycosylated GSL that suffer from low ionization. The derivatization in combination with UHPLC/MS or IM may also allow the separation of isomers by providing highly abundant diagnostic ions alongside a few by-product ions together with the increased sensitivity [105]. Besides decreasing the risk of false identification due to the presence of specific fragments, the derivatization can pose some challenges, e.g., possible contamination or formation of artifacts [36]. Various derivatization approaches for the structural elucidation of GSL isomers are discussed in more detail below.

#### Permethylation

MS/MS of permethylated GSL, where the active protons in –OH and –NH_2_ functional groups of GSL react with methyl iodide in alkaline solution (NaOH) of dimethylsulfoxide, is a powerful tool for rigorous glycan sequence and linkage determination, and ceramide composition due to the formation of specific fragments. However, the data analysis of permethylated GSL may be complicated since GSL may have different numbers of reaction sites. Permethylation can be carried out using both natural and isotope-labelled methyl iodide (i.e., ^12^CH_3_I, ^13^CH_3_I, or ^12^CD_3_I) [29]. When combined, the so-called differential isotope labelling technique is very useful in the quantitation of intact GSL, resulting in up to 20-fold signal enhancement compared to their low sensitivity in the presence of total lipid extracts. It has also been observed that alkaline conditions during permethylation significantly reduced ion-suppressing ester-linked lipids, thus encouraging the usefulness of permethylation for the quantitation of low-abundant GSL in complex mixtures [106]. On the contrary, the permethylation in NaOH/DMSO is not suitable for pH-sensitive functional groups of carbohydrates (e.g., O-acetylation) or GSL with polysialic acids, as these may be destroyed under such chemical conditions [31]. The use of permethylation in the structural analysis of GSL in the positive ion mode has already been demonstrated [107]. In addition, Ejsing et al*.* reported a shogun-based method for quantitative profiling of long-chain sphingoid base metabolites by using ^12^CD_3_I, which significantly enhanced the sensitivity [108].

#### Sialic acid-related derivatization strategies

Acidic GSL, such as gangliosides, include a large number of sialyl-linked glycan isomers with α2,3-, α2,6-, and α2,8-linked polysialic acids typically on the non-reducing ends of glycans [109]. Sialylated GSL are commonly analyzed in the negative ion mode, while they are hard to analyze in the positive ion mode due to their poor ionization efficiency [110]. It should also be stressed that MS cannot fully supply the glycan sequence since it has been well documented that various sialylated oligosaccharides lose sialic acid during in-source and post-source dissociation, decreasing the sensitivity of the molecular ion and dominantly yielding product ions lacking sialic acid, representing a major problem in MALDI-MS analysis of sialylated GSL [111, 112].

The distinct chemical derivatization methods of sialic acids, e.g., esterification [112], amidation [111], permethylation [113], or perbenzoylation [114], have been developed to stabilize the sialylated residues and to improve ionization efficiency of acidic GSL in the positive ion mode. This modification allows highly sensitive and simultaneous identification of both neutral and acidic GSL without ion mode switching, especially for gangliosides with more sialic acids [110]. Hanamatsu et al*.* [109] developed a method based on ring-opening aminolysis for discrimination between α2,3- and α2,8-linked sialic acid isomers of GSL-glycan by MS. Liu et al. [110] labelled the carboxyl group of sialic acid with an easily ionizable tertiary amine, i.e., N,N-dimethylethylenediamine (DMEN), which enhanced the ionization more than fourfold and provided a diagnostic ion to facilitate rapid structural assignments of gangliosides and discrimination of isomers. This strategy was successfully applied for the simultaneous identification of neutral and acidic GSL in human plasma. Moreover, the labelling approach using 2-(2-pyridilamino)-ethylamine (PAEA) has been developed by Huang’s group. The method has been applied to plasma using MRM-based LC/MS/MS method, which resulted in 15-fold enhancement of ionization compared to underivatized analogs. However, the derivatization efficiency of PAEA approach was not sufficient; thus, only monosialogangliosides (i.e., GM_1_, GM_2_, and GM_3_) were analyzed [115].

#### Isobaric labelling

Isobaric labelling is a relatively novel technique in glycosphingolipidomics, where samples are labelled with isobaric tags consisting of the same molecule with varying placement of isotopes (e.g., ^13^C and ^15^N). To date, two versions of commercially available isobaric tags have been applied to GSL, namely, iTRAQ™ (i.e., isobaric tag for relative and absolute quantitation) reacting with free amines [116] and aminoxyTMT™ (aminoxy tandem mass tag) [117] reacting with aldehyde and ketones.

#### Other derivatization strategies

There are also other derivatization strategies that can be used for GSL analysis. Peterka et al*.* [118] introduced highly reproducible derivatization of multiple lipid subclasses, including even simple GSL, using non-hazardous benzoyl chloride. Zheng et al*.* [119] developed a highly sensitive method for simultaneous analysis of multiple sphingoid bases using 3-(N,N-dimethylamino)propyl isothiocyanate (DMPI) and its isotopically labelled counterpart (d_4_-DMPI). A few other chemical strategies can be utilized for the elucidation of GSL structures, such as diluted sodium periodide oxidizing an OH group of sialic acid to aldehyde [120], 2,3-dichloro-5,6-dicyano-1,4-benzoquinone (DDQ) that transforms an OH group of a sphingosine into allyl ketone [121], or method called oxidative release of the neutral glycans (ORNG) by sodium hypochlorite converting a glycosidic bond of a ceramide to nitrile [122].

## Conclusions and outlook

GSL represent a diverse class of biomolecules that play crucial roles in biological processes and disease pathogenesis. Their identification and structural elucidation are pivotal for understanding their functions and metabolic pathways. MS has proven to be a powerful tool for GSL analysis due to its sensitivity and ability to provide detailed structural information on glycan sequences, linkage positions, and fatty acid composition. Although current MS approaches provide valuable insights into GSL structures, they often lack sufficient resolution to distinguish isomeric species with subtle structural differences. Moreover, the localization of carbon–carbon DB in the ceramide backbone poses a significant analytical challenge, as conventional dissociation techniques, such as the predominantly used CID, may not provide definitive information on the bond positions and their geometry. To address this issue, novel ion activation techniques, such as the Paternò-Büchi reaction, ozone-induced dissociation, or various types of epoxidation, have been developed and have shown great promise in locating carbon–carbon DB and determining their *cis/trans* geometry in GSL ceramides, thus overcoming a long-standing challenge in GSL analysis. In addition, other dissociation techniques, such as electron-mediated, photon-mediated, or radical-directed dissociation, may provide complementary insights into GSL structures, despite being less used. Another major limitation is the poor detection and characterization of highly complex and low-abundance GSL, which is hindered by their low ionization efficiency and incomplete or low extraction efficiency by classical extraction methods, as these GSL are more hydrophilic, thus restricting the discovery of novel GSL species. Looking ahead, further advances in MS instrumentation and ion mobility are expected to improve the sensitivity, selectivity, and throughput of GSL analysis. This will enable a comprehensive characterization of GSLs and their isomers in complex biological samples, paving the way for a deeper understanding of their role in health and disease and unlocking the full potential of GSL analysis to advance biomedical research.
